# *Xenopus Nkx6.3* Is a Neural Plate Border Specifier Required for Neural Crest Development

**DOI:** 10.1371/journal.pone.0115165

**Published:** 2014-12-22

**Authors:** Zuming Zhang, Yu Shi, Shuhua Zhao, Jiejing Li, Chaocui Li, Bingyu Mao

**Affiliations:** 1 State Key Laboratory of Genetic Resources and Evolution, Kunming Institute of Zoology, Chinese Academy of Sciences, Kunming, China; 2 Department of Clinical Laboratory, Children's Hospital of Chongqing Medical University, Chongqing, China; 3 Ministry of Education Key Laboratory of Child Development and Disorders; Key Laboratory of Pediatrics in Chongqing; Chongqing International Science and Technology Cooperation Center for Child Development and Disorders, Chongqing, China; 4 Kunming College of Life Science, University of Chinese Academy of Sciences, Kunming, China; University of Colorado, Boulder, United States of America

## Abstract

In vertebrates, the neural plate border (NPB) is established by a group of transcription factors including Dlx3, Msx1 and Zic1. The crosstalk between these NPB specifiers governs the separation of the NPB region into placode and neural crest (NC) territories and also their further differentiation. Understanding the mechanisms of NPB formation and NC development is critical for our knowledge of related human diseases. Here we identified Nkx6.3, a transcription factor of the Nkx family, as a new NPB specifier required for neural crest development in *Xenopus* embryos. *XNkx6.3* is expressed in the ectoderm of the neural plate border region at neurula stages, covering the epidermis, placode and neural crest territories, but not the neural plate. Inhibition of Nkx6.3 by dominant negative construct or specific morpholino leads to neural crest defects, while overexpression of Nkx6.3 induces ectopic neural crest in the anterior neural fold. In animal caps, Nkx6.3 alone is able to initiate the whole neural crest regulatory network and induces neural crest fate robustly. We showed that overexpression of Nkx6.3 affects multiple signaling pathways, creating a high-Wnt, low-BMP environment required for neural crest development. Gain- and loss-of-function of Nkx6.3 have compound effects on the expression of known NPB genes, which is largely opposite to that of Dlx3. Overexpression of Dlx3 blocks the NC inducing activity of Nkx6.3. The crosstalk between Nkx6.3, Dlx3 and Msx1 is likely crucial for proper NPB formation and neural crest development in *Xenopus*.

## Introduction

Neural crest (NC) cells are a multipotent, migratory cell population arising at the neural plate border (NPB) in vertebrates, which give rise to various cell lineages including craniofacial bones and cartilages, melanocytes and peripheral neurons [1]. Understanding the mechanisms of neural crest development is critical for our knowledge of related human diseases, including defects in pigmentation, craniofacial and heart development.

The development of neural crest is regulated by a multi-step gene regulatory network (GRN), involving complicated interactions of multiple signaling molecules and tissues [2]–[4]. The determination of NPB by a group of signaling molecules is the first step in neural crest development. During gastrulation, a mediolateral gradient of BMP activity is established in the ectoderm through the action of BMP antagonists diffusing from the underlying notochord, such that the medial ectoderm with low BMP activity develops into neural plate, and the lateral ectoderm with high BMP signal becomes epidermis [5]–[7]. The region in between with intermediate BMP activity will become the NPB region which is crucial for neural crest development [8]. Wnt and FGF signals play key roles to position the neural crest territory along the anterior-posterior axis [9]. Signals from neural and non-neural territory inhibit each other to sharpen and refine the NPB region [2], [10]–[15]. The signaling events that establish the neural plate border control the broad expression of a set of transcription factors, including members of the Zic, Pax, Dlx and Msx families [4], [13], [15]–[17]. These factors, known as neural plate border specifiers, further control the expression of a group neural crest specific genes, including *Snail1*, *Slug*, *FoxD3*, *Sox10*, *Sox9*, *AP-2* and *c-Myc* [4]. The neural crest specifier genes collectively control the expression of several downstream effector genes, which confer certain properties such as migration and multipotency before their terminal differentiation [18].

Another group of cells, the placodes, also originate from NPB, which are crucial for the development of the cranial sensory systems in vertebrates [19]. At early neurula stages, the neural crest territory occupies the medial side of NPB, within the trunk and head regions except the anteriormost neural folds [2]. The pre-placodal ectoderm, which expresses the panplacodal markers Six1 and Eya1, occupies the lateral part of the NPB, forming a “U” shape pattern at the anteriormost of head [20]. It has been proposed that neural crest and placodes develop from a common group of ancestor cells [19], which were then specified by different signals. However, recent studies support a binary competence model, according to which neural crest and placode originate differently from the neural and non-neural ectoderm respectively [21]. Dlx3, which is expressed in the placodal part of the NPB, is critical in the regulation of non-neural competence [13], [17], [21], [22]. Dlx and Msx can inhibit each other to determine the NPB cell identity, to become neural crest (Msx high and Dlx low) or placode (Msx low and Dlx high) [23]. However, the regulatory network to discriminate placode and neural crest fates and determine the sharp border between them remains elusive.

The Nkx family transcription factors are involved in a variety of developmental processes. Of the Nkx6 subfamily genes, *Nkx6.1* and *Nkx6.2* have been implicated in the control of cell differentiation in the central nervous system and pancreas [24]–[28]. *Nkx6.3*, a third member of this subfamily, is expressed in the anterior neural plate border region at neurula stages in *Xenopus* embryos [25]. Here, we analyzed the role of *Nkx6.3* in *Xenopus* neural crest development and NPB formation by gain and loss of function studies. We showed evidence that *XNkx6.3* is required for neural crest development and is able to induce neural crest fates dependent on Wnt signaling. Nkx6.3 is also involved in neural plate border formation and antagonizes the function of Dlx3.

## Results

### *XNkx6.3* is expressed in the neural plate border ectoderm

**Figure 1:**
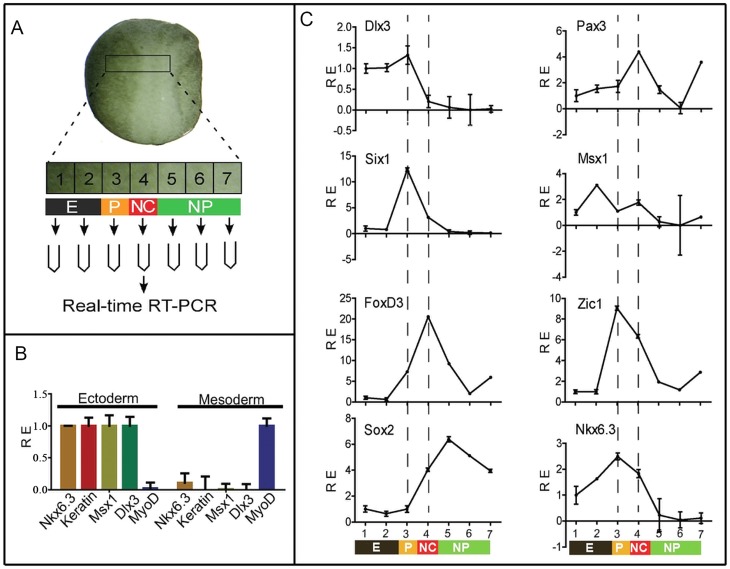
*XNkx6.3* is expressed in the neural plate border ectoderm. (A) Experimental strategy to verify the expression domain of *Nkx6.3* by qPCR. A transverse slice of tissue of the potential neural plate border region of single stage 17 embryos was dissected out and separated sequentially into 7 pieces, which were expected to represent epidermis, placode, neural crest and neural plate identities respectively. The explants were then processed to real-time RT-PCR analysis. E, epidermis; P, placode; NC, neural crest; NP, neural plate. (B) *Nkx6.3* is expressed predominantly in the ectoderm. A piece of tissue corresponding to region 3 in (A) was dissected out and separated into surface ectodermal and deep mesodermal parts. The expression of *Nkx6.3* and known ectodermal (*Keratin, Dlx3* and *Msx1*) and mesodermal (*MyoD*) genes were monitored by qPCR. (C) The expression of *Nkx6.3* and known neural plate border markers in a representative series of dissected epidermis, placode, neural crest, and neural plate explants from a single embryo at stage 17. The series of explants from each embryo were checked first for the expression of *Dlx3*, *Six1*, *FoxD3* and *Sox2*, and only those with relative clean separation of the epidermis, placode, neural crest and neural plate were further analyzed for the expression of *Nkx6.3* and additional markers. RE, relative expression.

By *in situ* hybridization, we showed previously that *XNkx6.3* is expressed in the non-neural ectoderm at cleavage to gastrula stages in *Xenopus*, and at neurula stages, its expression is gradually restricted to neural plate border regions [25]. However, due to its relative weak expression, we failed to verify its detailed expression in comparison to other neural plate border/neural crest markers using double *in situ* hybridization. We thus compared the expression patterns of *XNkx6.3* with other neural plate border/neural crest markers in serial dissected pieces of ectodermal tissues along the medial-lateral axis of the neural plate border regions by real-time RT-PCR. A transverse slice of tissue of the neural plate border region of single stage 17 embryos was dissected out and separated sequentially into 7 continuous pieces, which were then proceeded to real-time RT-PCR analysis. The 7 pieces of tissues were expected to represent epidermis, placode, neural crest and neural plate identities respectively (Fig. 1A). We first checked whether *XNkx6.3* is expressed in the ectodermal or mesodermal tissues. A piece of tissue corresponding to the placode/neural crest region was dissected out and separated into surface ectodermal and deep mesodermal parts. The identities of the tissues were confirmed by the relative expression levels of known epidermis marker *keratin* and mesodermal marker *myoD* (Fig. 1B). As expected, *Dlx3* and *Msx1*, two genes involved in neural plate border formation, were predominantly expressed in the ectodermal regions. In such experiments, the expression of *Nkx6.3* was always found to be expressed predominantly in the ectodermal part and only very weakly if any in the mesoderm.

The expression of *XNkx6.3* was then examined in the dissected neural plate border tissues using real-time PCR (Fig. 1C). To verify the success of the separation of the tissues, the expression known markers of different tissues, *Dlx3* for epidermis and placode, *Six1* for placode, *FoxD3* for neural crest and *Sox2* for neural plate, were first examined in such tissue serials. In successful series, the expression of these markers peaked in different pieces of tissues as expected, such that *Dlx3* was expressed in the epidermis (pieces 1–2) and placode (piece 3), *Six1* and *FoxD3* peaked in the placode (piece 3) and neural crest (piece 4) respectively, and *Sox2* was detected in the neural plate (pieces 5–7) and neural crest region (piece 4), but not in the placodal region (piece 3) (Fig. 1C). Of the neural plate border specifiers, *Pax3* and *Zic1* are both highly expressed in the placode and neural crest regions, peaked in the neural crest and placode regions respectively. *Msx1* was found to be expressed in the epidermis and neural crest regions, and at a weaker level in the placode. In such a serial of tissues, *XNkx6.3* was found to be mainly expressed in the placode and neural crest regions, and to a weaker extent, also in the epidermal regions (Fig. 1C).

### *Nkx6.3* is required for neural crest development

**Figure 2:**
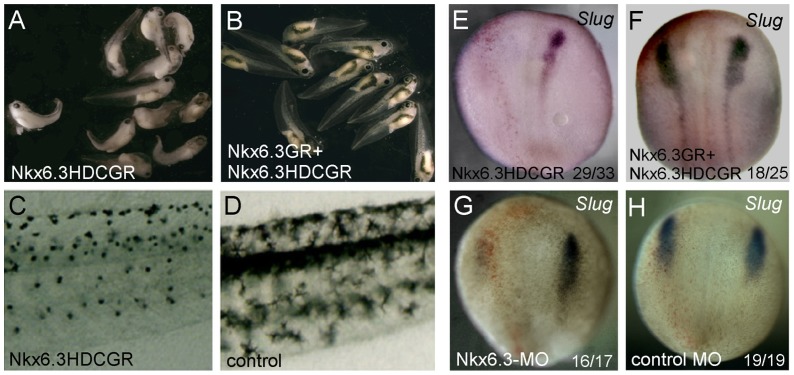
Nkx6.3 is required for neural crest development in *Xenopus*. Injection of a inducible dominant negative form of Nkx6.3, Nkx6.3-HDC, induced severe defects in pigmentation at tadpole stages (A) when dexamethasone was added, which was rescued by co-expression of the wild type Nkx6.3-GR construct (B). The pigmentation patterns of the trunk region of an Nkx6.3-HDC injected embryo and a control embryo were highlighted in (C) and (D) respectively. Injection of *Nkx6.3HDC-GR* reduced the expression of *Slug* (E), which can be restored by co-injected *Nkx6.3GR* mRNA (F). (G) and (H) Injection of specific morpholino against *Nkx6.3* but not a control morpholino impaired the expression of the neural crest marker *Slug*. The injected sides in (E)–H) are on the left, labeled by the red staining of the co-injected tracing lacZ. In (E)–H), the numbers of embryos showing similar changes of gene expression and total injected embryos in each group are indicated.

Nkx6 proteins are believed to act as transcriptional repressors with its eh1 domain as the repressor domain [29]. We constructed an eh1 deletion construct (the HDC construct) which should lack repressor activity and antagonize the function of endogenous *Nkx6.3*. As injection of wild type *Nkx6.3* or the HDC-*Nkx6.3* generally leads to gastrula defects, we constructed glucocorticoid receptor (GR) fusion constructs of *Nkx6.3* and HDC, the nuclear translocation and activity of which could be induced by addition of dexamethasone (DEX). When induced after gastrulation stage (stage 12), embryos injected with the HDC construct showed severe defects in pigmentation at tadpole stages (Fig. 2A, 2C), a hallmark of neural crest development. This effect was nicely rescued by co-expression of the *Nkx6.3*-*GR* construct (Fig. 2B). At early neurula stages, the expression of neural crest marker *Slug* was abolished in *HDC*-*Nkx6.3* injected embryos (Fig. 2E), but not when *Nkx6.3-GR* mRNA was co-expressed (Fig. 2F).

We also tried to block the function of endogenous *Nkx6.3* using one specific morpholino (MO) against its ATG start cordon region. We confirmed that the MO efficiently blocked the expression of a reporter GFP construct harboring the targeted sequence at its 5′ ATG region (data not shown). Embryos injected with the *Nkx6.3* MO but not the control MO showed impaired expression of the neural crest marker *Slug* (Fig. 2G, 2H), supporting a role of *Nkx6.3* in neural crest development. However, the effect of the *Nkx6.3* MO on neural crest induction was poorly rescued by co-injection of either wild type *Nkx6.3* or the *Nkx6.3*-*GR* (induced at stage 11–12). This could be due to the fact that *Nkx6.3* actually inhibits NC development when injected into the NC territory itself (see below) and when induced by DEX, the transient nuclear Nkx6.3-GR level would be much higher than just compensating the loss of endogenous Nkx6.3.

### *Nkx6.3* is able to induce ectopic neural crest dependent on Wnt signaling

**Figure 3:**
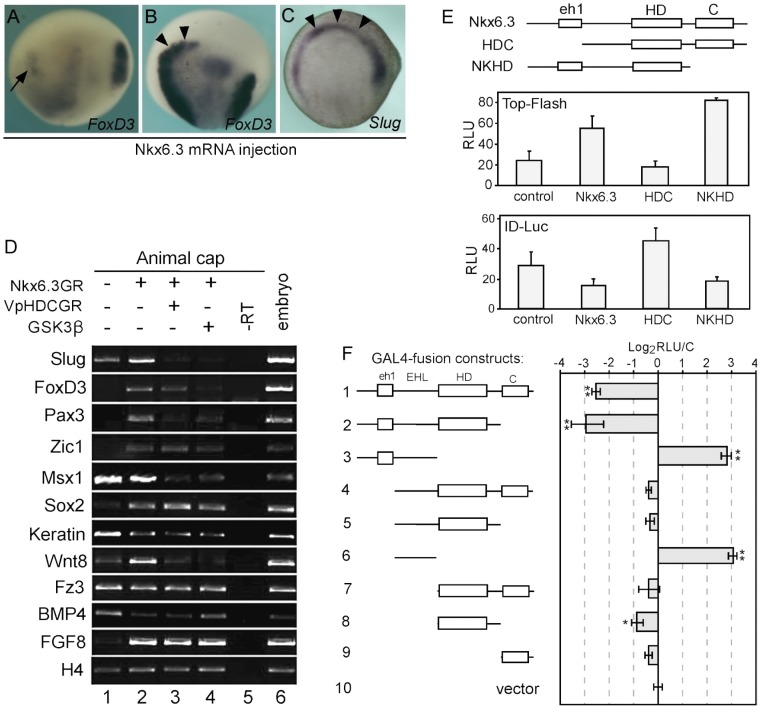
Nkx6.3 is able to induce neural crest dependent on Wnt signaling. (A) *Nkx6.3GR* mRNA was injected into one blastomere at 2-cell stage and DEX was added at stage 11, the expression of neural crest marker *FoxD3* was inhibited on the injected side (arrow). (B, C) Local injection of *Nkx6.3* mRNA at 32-cell stage induced neural crest (arrowheads) in the anterior neural folds. (D) In animal caps, overexpression of *Nkx6.3* induced the expression of neural crest markers and that of Wnt8 and FGF8 (lanes 1, 2). The VpHDC construct, in which the eh1 repressor domain was replaced by the Vp16 activation domain, repressed the neural crest inducing effect of *Nkx6.3* (compare lanes 2, 3). Inhibiting Wnt signaling by GSK3β blocked most of the effects of *Nkx6.3* on marker gene expression (lane 4). (E) The effect of different *Nkx6.3* constructs on the expression of luciferase reporter genes of Wnt (Top-Flash, middle panel) and BMP (ID-Luc, lower panel) signaling in *Xenopus* embryos. The domain structures of *Nkx6.3* and the HDC, NKHD constructs were shown in the upper panel. eh1, eh1 repressor domain; HD, homeodomain; C, C-terminal domain. (F) Effects of the fusion constructs of GAL4 and various *Nkx6.3* domains on the expression of a GAL4 luciferase reporter gene. Wild type *Nkx6.3* and that lacking the C terminal domain worked as repressors (bars 1, 2) while the EHL region with or without the eh1 domain both activated the transcription of the reporter (bars 3, 6). EHL, the linker region between the eh1 repressor domain and the HD domain. *, p<0.05; **, p<0.01. The expression of the different constructs were confirmed by Western blot (data not shown).

As Nkx6.3 is required for neural crest development, we next tested whether overexpression of Nkx6.3 is sufficient to induce ectopic neural crest. However, when *Nkx6.3-GR* was injected at 2-cell stage and DEX was added at stage 11, the neural crest marker expression was rather inhibited than induced (Fig. 3A). We then tried injections at 32-cell stage, targeting the dorsal neural plate region. Interestingly, when the anterior neural fold regions were targeted, ectopic neural crest was frequently observed (Fig. 3B, 3C). We further tested the neural crest induction activity of *Nkx6.3* in animal cap explants. Interestingly, in animal caps, *XNkx6.3* alone is sufficient to induce the expression of the whole panel of neural crest genes, including *Zic1*, *Pax3*, *Slug* and *FoxD3* (Fig. 3D).

During development, the presumptive neural crest territory is induced at the neural plate border through the interplay of different signaling pathways including BMPs, Wnts and FGFs. We tested whether components of these signaling pathways were attenuated in *Nkx6.3* injected animal cap explants. The results showed that overexpression of *Nkx6.3* strongly induced the expression of *Wnt8* and *FGF8* while inhibited that of *BMP4* in animal caps (Fig. 3D). The expression of the Wnt receptor, *Frizzled 3*, which has also been shown to regulate neural crest development [30], did not change. We then tested the effect of different *Nkx6.3* constructs on Wnt and BMP signaling using reporter assays in *Xenopus* embryos (Fig. 3E). Consistent with above results, wild type *Nkx6.3* activates the Wnt reporter expression while inhibiting the BMP reporter expression. The HDC construct, which lacks the eh1repressor domain, shows the opposite effects, inhibiting Wnt while activating BMP signaling. The NKHD construct, which lacks the C-terminal domain, works similarly to wild type *Nkx6.3* (Fig. 3E). These data suggest that overexpression of *Nkx6.3* in animal caps attenuated the signaling environment to promote neural crest development.

In animal caps, the induction of neural crest genes is dependent on Wnt signaling, since co-expression of a Wnt signaling inhibitor, *Gsk3β*, completely abolished its activity on neural crest induction (Fig. 3D). Interestingly, the expression of *FGF8* was also strongly induced by *Nkx6.3*, which can not be blocked by *GSK3β*. Also, the VpHDCGR construct, in which the eh1 repressor domain of *Nkx6.3* was replaced by the VP16 activation domain, effectively blocked most of the activities of *Nkx6.3* on neural crest genes expression in animal caps, yet it failed to repress the induction of *FGF8* (Fig. 3D). These results suggested that *Nkx6.3* works mainly as a transcriptional repressor to induce neural crest genes expression, including that of *Wnt8*. However, it likely works as an activator to stimulate the expression of *FGF8*. To check the possibility that *Nkx6.3* is able to work both as a transcriptional repressor and an activator, we tested the activities of a series of fusion constructs of *Nkx6.3* deletions with GAL4 DNA binding domain on the expression of a luciferase reporter harboring GAL4 binding sites (Fig. 3F). In the reporter assay, full length Nkx6.3 and the NKHD construct effectively inhibited the reporter expression, while the construct containing only the linker region between the eh1 repressor domain and the HD domain (EHL, #6 in Fig. 3F) works as a transcriptional activator. The construct containing the eh1 domain and the EHL region (#3 in Fig. 3F) also activates the reporter expression while the other constructs most showed very weak repressor activities. Thus *Nkx6.3* is potentially able to work as either a repressor or an activator in different contexts.

**Figure 4:**
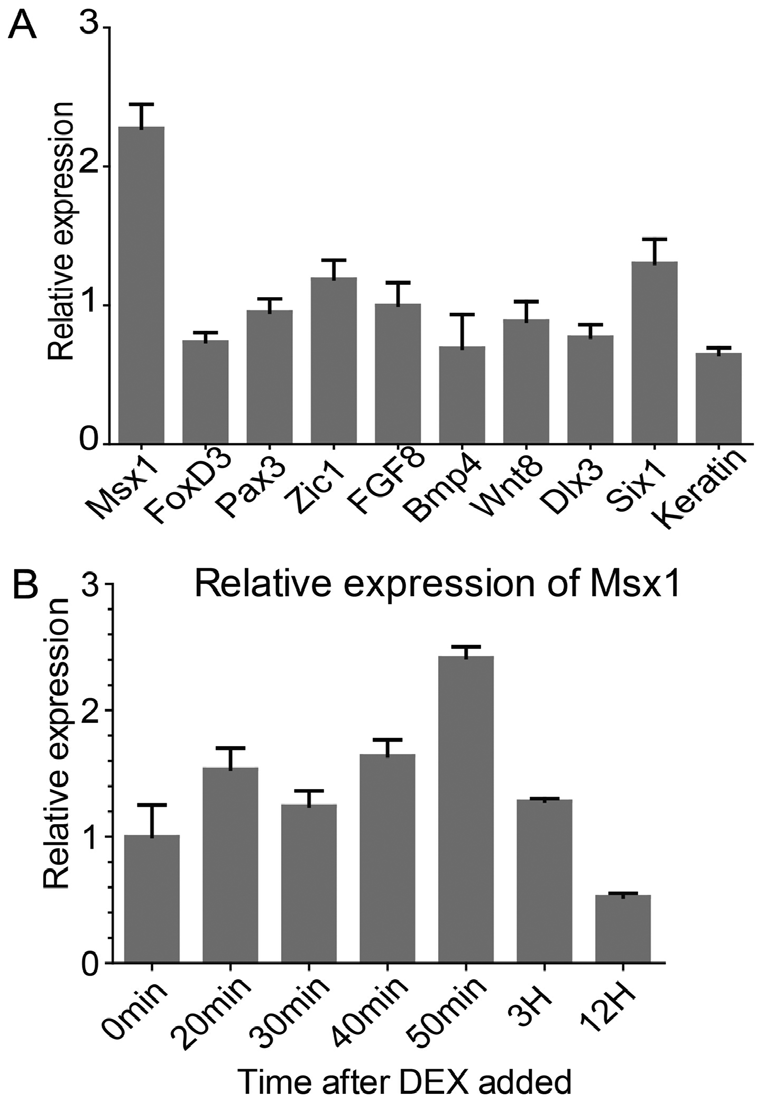
*Msx1* is an immediate target gene in response to Nkx6.3. (A) 1 ng *Nkx6.3*GR mRNA was injected into each cell at 2-cell stage. Animal caps were dissected at Stage 9, treated with cycloheximide for 30 minutes to inhibit protein synthesis, and then cultured in media containing dexamethasone for 2 hours before processed for RT-qPCR to monitor the expression of the marker genes indicated. Among the tested genes, *Msx1* was the only one up-regulated clearly under such condition. (B) Dynamic induction of *Msx1* by *Nkx6.3*. The induction of *Msx1* was monitored at different time points after induction as described in (A). The expression level of *Msx1* was up-regulated in one hour, but was then down-regulated at later stages.

In order to find the immediate-early response genes to Nkx6.3, we performed RT-qPCR to test the expression of these genes in animal caps treated with cycloheximide to block protein synthesis. In the presence of cycloheximide, after addition of dexamethasone, the induction of most of the neural crest genes was clearly blocked. However, the expression of *Msx1* remained stimulated to more than 2 folds (Fig. 4A). However, in our previous semi-quantitative analysis, the induction of *Msx1* by *Nkx6.3* was less clear (Fig. 3D, lane 2). We then tested the time course of *Msx1* induction in *Nkx6.3* injected animal caps (Fig. 4B). Our results showed that the induction of *Msx1* was transient, which went up in 1 hour but then declined to control level in about 3 hours. As the two genes are co-expressed in the neural plate border region (Fig. 1), we suggest that *Msx1* is potentially one of the direct targets of Nkx6.3.

### *Nkx6.3* is a neural plate border specifier

The above data showed that at least in animal cap assays, *Nkx6.3* is able to modulate Wnt, BMP and FGF signaling, which are required for proper neural border formation *in vivo*. We then tested systematically the effects of *Nkx6.3* overexpression and inhibition on the expression of neural plate border genes as well as the placode and neural crest marker genes.

**Figure 5:**
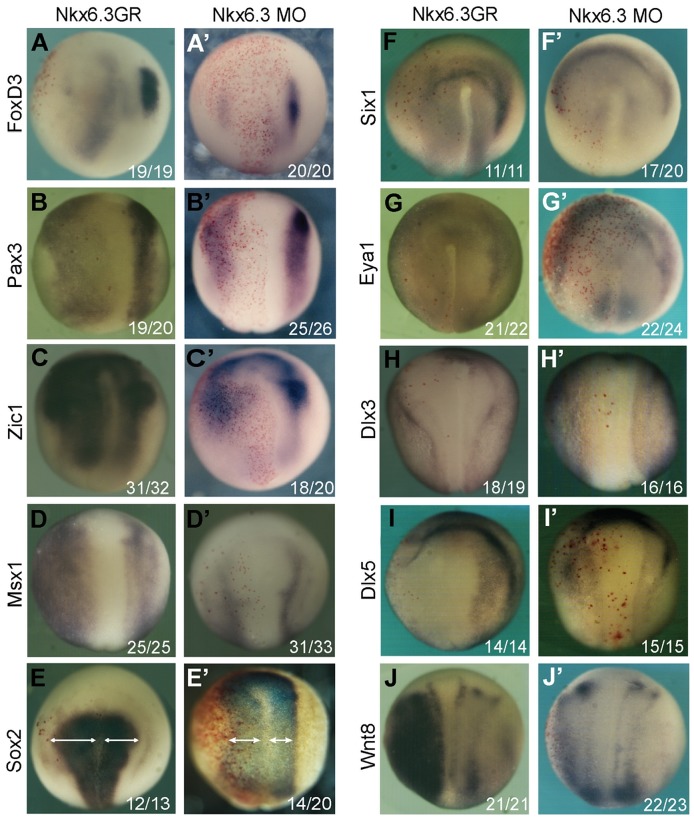
Nkx6.3 is a neural plate border specifier. The effects of *Nkx6.3* overexpression (A–J) or knockdown (A′–J′) on the expression of indicated neural and non-neural ectodermal markers and that of *Wnt8*. The injected sides were all on the left marked by red β-galactosidase staining. In (E) and (E′), the arrowed lines indicate the width of the neural plates. The numbers of embryos showing similar changes of gene expression and total injected embryos in each group are indicated.

In the overexpression experiments, the embryos were injected with *Nkx6.3*GR mRNA at 4-cell stage, induced at the end of stage 11 and processed for *in situ* hybridization at stage 15. As mentioned above, overexpession *Nkx6.3* strongly inhibited the expression of the neural crest marker *FoxD3* (Fig. 5A). Consistent with the results in animal cap assays, Nkx6.3 also stimulated the expression of neural plate border specifiers *Zic1* and *Msx1*(Fig. 5C, 5D). The expression level of *Pax3*, however, was clearly reduced in *Nkx6.3* injected sides (Fig. 5B), although its expression domain was expanded. The expression of the pan neural marker *Sox2* expanded slightly (Fig. 5E). The expression of the panplacodal markers *Six1* and *Eya1*, general nonneural markers *Dlx3* and *Dlx5*, was all reduced in *Nkx6.3* injected areas (Fig. 5F–I), suggesting a general failure of neural plate border development. Interestingly, as in animal caps, overexpression of *Nkx6.3* induced strong *Wnt8* expression in injected sides (Fig. 5J), which might be partially responsible for the patterning defects of the neural plate border.

When *Nkx6.3* was blocked by specific morpholino, the expression levels of the neural crest markers *FoxD3*, neural plate border specifier *Pax3*, *Zic1* and *Msx1* were all reduced (Fig. 5A′–D′), with the expression domain of *Pax3* and *Zic1* expanded. The expression of *Sox2* also expanded slightly (Fig. 5E′). In the *Nkx6.3* morphants, the placode markers *Six1* and *Eya1* became slightly stronger (Fig. 5F′, 5G′). Interestingly, the expression border of *Dlx3* and *Dlx5* became blurred when *Nkx6.3* was knocked-down (Fig. 5H′, 5 I′). The expression of *Wnt8* had no clear change in *Nkx6.3* morphants (Fig. 5J′).

**Figure 6:**
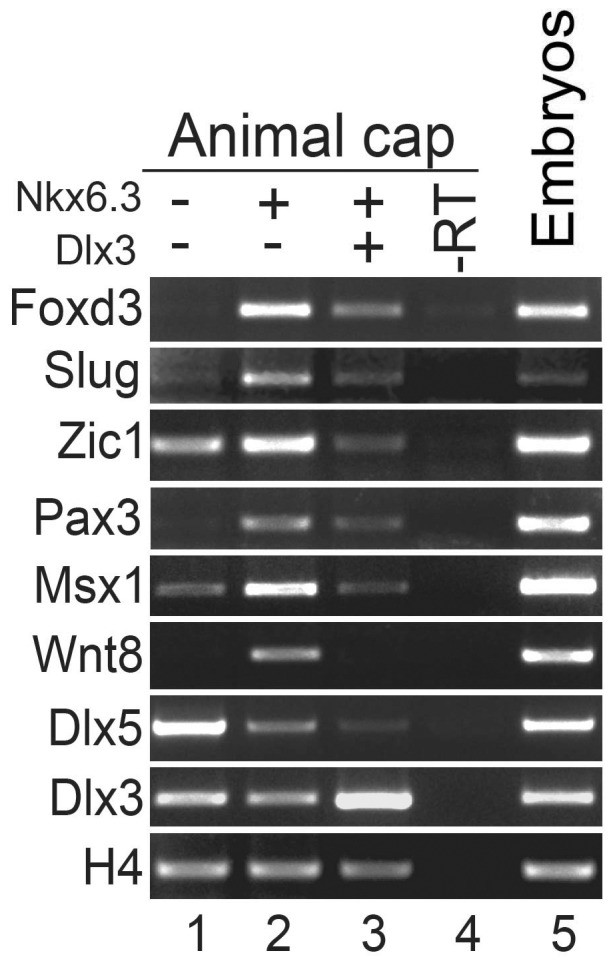
Dlx3 blocks the neural crest induction activity of Nkx6.3. RT-PCR analysis in animal cap assay showing the effect of *Dlx3* on the expression of the indicated neural crest genes induced by *Nkx6.3*. Note that the expression of endogenous *Dlx3* and *Dlx5* were all reduced upon *Nkx6.3* over-expression. -RT, negative control with reverse-transcriptase omitted in the RT reaction; embryo, RNA template from whole embryos was used as a positive control.

The above gain- and loss-of-function phenotypes of *Nkx6.3* are largely opposite to that of *Dlx3* [21], suggesting opposite roles of the two genes in neural plate border development. We then tested whether *Dlx3* could inhibit the neural crest induction activity of *Nkx6.3*. Indeed, co-expression of *Dlx3* largely inhibited the activity of *Nkx6.3* to induce neural crest markers in animal cap assay (Fig. 6). Interestingly, overexpression of *Nkx6.3* also reduced the expression of endogenous *Dlx3* and *Dlx5* (Fig. 6). These data suggest that *Nkx6.3* and *Dlx3* have opposite roles as regards to neural crest development. *Dlx3* is expressed in the placodal region where *Nkx6.3* is co-expressed. *Dlx3* has been suggested to regulate the non-neural competence [13], [21] and we suggest that the function of *Dlx3* is dominant *in vivo* in the placodal region. In the neural crest territory, *Nkx6.3* but not *Dlx3* is expressed (Fig. 1), where it functions to regulate the signaling environment to promote neural crest development.

## Discussion

### *Nkx6.3* is required for neural crest development

In this study, we provided several lines of evidence that *Nkx6.3* is required for neural crest development in *Xenopus*. First, *Nkx6.3* is expressed in the ectoderm of the neural plate border region at neurula stages, covering the epidermis, placode and neural crest territories, but not the neural plate (Fig. 1). Second, inhibition of *Nkx6.3* either by dominant negative construct or specific morpholino leads to neural crest defects, including pigmentation defects and loss of neural crest marker expression (Fig. 2). Third, overexpression of *Nkx6.3* promotes ectopic neural crest development in the anterior neural fold, although it inhibits endogenous neural crest when injected at the NC territory itself (Fig. 3). Furthermore, in animal caps, we showed that *Nkx6.3* alone is able to initiate the whole neural crest regulatory network and induce neural crest fate robustly. In animal caps, *Nkx6.3* strongly induced the expression of *Wnt8* and *FGF8* while inhibited that of *BMP4*, thus created a high-Wnt, low-BMP environment required for neural crest development. We confirmed that *Nkx6.3* overexpression also induces robust *Wnt8* expression in whole embryos (Fig. 5J), which has been shown to be a NC inducer [9]. We showed that *Nkx6.3* mainly works as a transcriptional repressor in neural crest induction, as an activator version of *Nkx6.3* (VpHDC) works as a dominant negative form in this process (Fig. 3D). Thus its induction of *Wnt8* is most likely indirect. As *Nkx6.3* has a compound effect on various signaling pathways, its overexpression in the neural crest territory itself likely interferes with the signaling environment required for NC development and thus inhibits NC induction. Active Wnt signaling is required for *Nkx6.3* in neural crest induction, as co-expression of Gsk3β, a Wnt signaling inhibitor, abolished its activity on neural crest induction. Although in animal caps, *Nkx6.3* is able to stimulate *FGF8* expression likely as a transcriptional activator, it can not in the presence of protein synthesis inhibitor, suggesting its effect is also indirect. The only potential direct gene of *Nkx6.3* implicated from our study is *Msx1*, which was still activated in the presence of cycloheximide. Interestingly, the induction of *Msx1* by *Nkx6.3* was transient, which went down to control level in about 3 hours. As *Msx1* is an established target of BMP signaling, we assume that this effect is likely a feedback of the inhibition of BMP signaling by *Nkx6.3* at later stages.

### *Nkx6.3* as a neural plate border specifier

Its expression pattern and gain- and loss-of function effects on NPB formation support *Nkx6.3* as a new neural plate border specifier. *Nkx6.3* is expressed in the non-neural ectoderm and neural crest territory at the neural plate border of neurula stage embryos (Fig. 1). Overexpression of *Nkx6.3* upregulates while loss of function of *Nkx6.3* reduces the expression of the NPB genes *Zic1* and *Mxs1* (Fig. 5C, 5D). However, either overexpression or inhibition of *Nkx6.3* leads to reduced expression of *Pax3*, although its expression domain became expanded (Fig. 5B). This could be the reason that *Nkx6.3* overexpression inhibits rather than induces NC development when injected in the NC territory (Fig. 2A).

Interestingly, the gain- and loss-of-function phenotypes of *Nkx6.3* on NPB markers are largely opposite to that of *Dlx3* [21], which is expressed in the placodal part of the NPB and is critical for placode development. *Nkx6.3* and *Dlx3* seem to promote placode and neural crest development respectively and antagonize the function of each other when overexpressed. Indeed, co-expression of *Dlx3* largely inhibited the NC induction activity of *Nkx6.3* assay (Fig. 6). When injected into the NC domain, *Nkx6.3* also inhibits the expression of neural crest as well as neural plate border marker genes [21]. On the other hand, overexpression of *Nkx6.3* reduced the expression of *Dlx3* and *Dlx5* and also the neural placodal markers *Six1* and *Eya1* (Fig. 5F–I). *Dlx3* has been shown to work as a transcription activator to repress neural fates [13] and is able to repress Wnt-β-catenin signaling when overexpressed [31]. Thus it is possible that *Nkx6.3* and *Dlx3* might share common target genes or regulatory units. Indeed, we also observed activation of *Wnt8* expression in *Dlx3* morphants (data not shown), as in the case of *Nkx6.3* overexpression. *Nkx6.1*, which contains a homeodomain highly similar to that of *Nkx6.3*, has been shown to bind elements containing the homeodomain core-binding site (5′-TAAT-3′or 5′-ATTA-3′) [32], [33], similar to that of *Dlx3* [34]. Thus it is possible that the *Nkx6.3* and *Dlx3* might directly compete for common target gene regulation.

Another possibility is that *Nkx6.3* inhibits the function of *Dlx3* indirectly through induction of *Msx1*, which has been shown to antagonize *Dlx3* through forming heterodimers with it [35]. In zebrafish, the mutual antagonism between Msx and Dlx proteins has also been shown to be required for normal placode development [23]. In mouse, *Msx1* is able to repress the pre-placodal marker *Six1* through direct binding to its enhancer while the binding of *Dlx5* activates it [36]. Thus the stimulation of *Msx1* by *Nkx6.3* might account for its activity to inhibit placodal development. *Nkx6.3* and *Msx1* are both co-expressed with *Dlx3* in the placodal region and might contribute to the fine tuning of the signaling environment for placode development *in vivo*. However, other factors must be involved in its induction of NC fates, since *Nkx6.3* works mainly as a repressor to induce NC and that overexpression of *Msx1* expands NC development in whole embryos [16], unlike *Nkx6.3*.

In summary, our study established *Nkx6.3* as a neural plate border specifier required for neural crest development. Together with *Dlx3* and *Msx1*, it is likely involved in the regulation of local signaling environment for proper NPB formation and also downstream events of neural crest development.

## Materials and Methods

### Ethics Statement

The care of *Xenopus laevis* (Nasco), *in vitro* fertilization procedure and embryos study were performed according to protocols approved by the Ethics Committee of Kunming Institute of Zoology, Chinese Academy of Sciences (permit number: SYDW20070301001).

### Microinjection and *in situ* hybridization

*In vitro* fertilization, embryo culture, whole mount *in situ* hybridization,preparation of mRNA, and microinjection were carried out as described [37]. The sequence of the antisense morpholino oligo (MO) for *XNkx6.3* used was: 5′- TAGGCCTTCTGCTCTCTCAACATGG -3′, which was obtained from Gene Tools (OR). A standard control oligo was used as a negative control morpholino which targets a human beta-globin intron mutation (Gene Tools). For *in situ* hybridization, the probes for *Slug, FoxD3, Pax3, Zic1, Sox2, Msx1, Six1, Eya1, Dlx3, Dlx5* and *Wnt8* were used as described [11], [16], [38]–[42].

### RNA isolation and reverse transcriptase PCR assay

Total RNAs were extracted using the Trizol total RNA extract kit (Tiangen) and reverse transcribed using the Fermentas RevertAid First Strand cDNA Synthesis Kit to prepare templates for semi-quantitative or real-time quantitative PCR (qPCR).

For traditional RT-PCR analysis, the primers for *Pax3*, *Slug*, *Msx1*, *Zic1*, *FoxD3* and *FGF8* and *Fz3* were used as described [8], [43]–[45]. Primers for *XWnt8* were: forward primer: 5′GACAAGATGCCAGAGCCCTAA; reverse primer: 5′TAAGTTCAGACCCGGCCACA. H4 was used as a loading control. The Qiagene QuantiNova probe PCR kit was used to monitor the expression of *Nkx6.3* and the reference GAPDH by probe method. The primers and probes used were: *Nkx6.3*: forward primer: 5′CCCATCATCCTGGAGCATTT; reverse primer: 5′TGGCATCCAGAAGATTTCATTTC; probe: 5′TGCTCCCATCCTACTC, labeled with FAM and MGB; GAPDH: forward primer: 5′ GTCTGGCTCCTCTCGCAAAG, reverse primer: 5′GTCATGAGTCCCTCAACAATGC; Probe: 5′TCATCAACGACAACTTT, labeled with VIC and MGB. The primers and probes were synthesized by Invitrogen. The expression of other genes was examined by SYBR green qPCR using the following primers:

*Dlx3*F: 5′ TCGGCCGTTTGTCCATTACA 3′, R: 5′GGTTTCGGGCTCTTCCTTCA 3′; *Wnt8*F: 5′GTCGGGTAACAGTGCTGACA3′, R: 5′ATAAGTTCAGACCCGGCCAC3′;

*Six1*F:5′ CTTACTCCCTGAGCGCACTT 3′, R: 5′ GGTCGCTCTTACGATCCCAG 3′.

The primers for GAPDH, Keratin, MyoD, Six1, Sox2, FoxD3, Pax3, Zic1, Msx1 and Bmp4 were used as described [15], [46]–[48]

### Plasmids construction

The full open reading frame of X*Nkx6.3* and that without the eh1 domain coding region were cloned into pCS2-GR vector to create the Nkx6.3GR and Nkx6.3HDCGR constructs. The VpHDCGR construct was prepared by cloning the HDCGR fragment into a VP16 expression vector [49]. Different fragments X*Nkx6.3* were cloned into the pBIND vector (Promega), which contains the yeast GAL4 DNA-binding domain, for the expression of GAL4 fusion proteins with different X*Nkx6.3* domains.

### Luciferase reporter assays

For luciferase reporter assay in *Xenopus* embryos to monitor the effect of *Nkx6.3* on Wnt signaling, the reporter plasmids (25pg of TOP-flash and 5pg of pTK-renilla) and 0.5ng mRNAs of different *XNkx6.3* constructs (*Nkx6.3GR*, *VpHDCGR* and *NKHDGR*) were injected into animal poles of all blastomeres at 4-cell stage. dexamethasone was added immediately after injection to activate of the *Nkx6.3* constructs. The embryos were harvested at Stage 10, divided into 3 groups (>10 embryos each group), lysed and analyzed using the Dual-Luciferase Reporter Assay System (Promega). The effect of *Nkx6.3* on BMP signaling was examined in animal caps using the ID-reporter (gift from Prof. Jing). The reporter plasmids (25pg of ID-reporter and 5pg of pTK-renilla) and 0.5 ng *Nkx6.3GR* mRNA were injected into animal poles of all blastomeres at 4-cell stage and cultured in media containing dexamethasone. Animal caps were cut at Stage 9, cultured till Stage 12 and lysed for reporter activity measurement. The control embryos were cultured without dexamethasone.

To test the transcriptional activity of various *XNkx6.3* constructs, HEK293T cells in 96-well plates were transfected with the pG5-Luc (100ng) which contains GAL4 binding sites in its promoter region and pRL-TK (10ng) reporters (Promega) and the GAL4-fusion *Nkx6.3* constructs in pBIND. The luciferase activities were analyzed 24 hours after transfection using the Dual-Luciferase Reporter Assay System (Promega). Statistical significance test was done using Student's t-test.
